# Cardiac Transplantation Does Not Improve Exercise Tolerance, Muscle Mass, or Substrate Metabolism in Barth Syndrome

**DOI:** 10.1002/jmd2.70034

**Published:** 2025-07-07

**Authors:** W. Todd Cade, Kathryn L. Bohnert, Linda R. Peterson, Lisa de las Fuentes, Emily Poehlein, Cynthia L. Green, Christina A. Pacak, Barry J. Byrne, Dominic N. Reeds, Adil Bashir, Brian Feingold, Carolyn Taylor

**Affiliations:** ^1^ Doctor of Physical Therapy Division Duke University School of Medicine Durham North Carolina USA; ^2^ Program in Physical Therapy Washington University School of Medicine St. Louis Missouri USA; ^3^ Department of Medicine Washington University School of Medicine St. Louis Missouri USA; ^4^ Department of Biostatistics & Bioinformatics Duke University School of Medicine Durham North Carolina USA; ^5^ Department of Neurology University of Minnesota School of Medicine Minneapolis Minnesota USA; ^6^ Department of Pediatrics University of Florida School of Medicine Gainesville Florida USA; ^7^ Department of Electrical and Computer Engineering Auburn University Auburn Alabama USA; ^8^ Department of Pediatrics (Cardiology) University of Pittsburgh School of Medicine Pittsburgh Pennsylvania USA; ^9^ Department of Pediatrics Medical University of South Carolina Charleston South Carolina USA

**Keywords:** Barth syndrome, cardiac transplantation, exercise intolerance, mitochondrial dysfunction, skeletal muscle energetics, substrate metabolism

## Abstract

Barth syndrome (BTHS) is a rare X‐linked recessive disorder characterized by mutations in the TAFAZZIN gene, leading to mitochondrial dysfunction, cardioskeletal myopathy, neutropenia, exercise intolerance, and growth delay. While cardiac transplantation can improve heart function in BTHS patients, the metabolic effects of this procedure remain poorly understood. This study aimed to compare skeletal muscle morphology, substrate metabolism, energetics, and exercise tolerance in individuals with BTHS who had undergone cardiac transplantation (BTHS‐T) versus BTHS patients without transplantation (BTHS‐noT) and healthy controls. Six (*n* = 6) BTHS‐T participants (3 adolescents, 3 adults) were compared with *n* = 29 BTHS‐noT and *n* = 28 healthy controls. All participants underwent graded exercise testing, echocardiography, body composition analysis, and clinical metabolism studies, including ^31^P‐MRS to assess mitochondrial energetics. No significant differences were observed in fat‐free mass between BTHS‐T participants and BTHS‐noT. Exercise capacity (V̇O_2peak_) was significantly lower in both BTHS groups compared to controls, with lower heart rate responses during peak exercise. In addition, plasma lactate was higher in both BTHS groups compared to healthy controls. Skeletal muscle energetics showed slower phosphocreatine recovery and reduced ATP production in BTHS participants, regardless of cardiac transplantation status. Findings suggest while cardiac transplantation in BTHS may improve heart function, it may not normalize skeletal muscle mass and energetics, metabolic abnormalities, and exercise intolerance. The study enhances our understanding of long‐term metabolic consequences of BTHS and potential limitations of cardiac transplantation in ameliorating these dysfunctions. Further research is needed to explore targeted therapies to address underlying metabolic abnormalities in BTHS patients.

**Summary**

- Cardiac transplantation in Barth syndrome does not improve exercise tolerance, body composition, or skeletal muscle engergetics and metabolism therefore interventions targeting skeletal muscle abnormalities in Barth syndrome are needed.

## 1 Introduction

Barth syndrome (BTHS) is an ultra‐rare X‐linked recessive condition caused by mutations in the tafazzin gene (*TAFAZZIN*) resulting in abnormal cardiolipin remodeling in mitochondria [1]. Alterations in cardiolipin remodeling manifest as smaller and fragmented mitochondria [2], disruptions in supercomplex formation [3, 4], inner mitochondrial membrane instability [4] and reduced respiratory capacity [5]. Clinical manifestations of BTHS include heart failure, skeletal myopathy, exercise intolerance, neutropenia, and growth delay [6, 7].

Treatment of cardiomyopathy in BTHS is not optimal, and approximately 15% of BTHS ultimately require cardiac transplantation [8, 9]. Cardiac transplantation in patients *without* inherited metabolic disease, including BTHS, improves muscle mass [10], skeletal muscle oxidative capacity [11], vascular function [12, 13], and exercise tolerance [13, 14, 15, 16, 17] and can cause a variety of changes in skeletal muscle metabolism including a shift towards improvements in oxidative and glycolytic enzymes [18]. We have previously demonstrated severely impaired exercise tolerance, low skeletal muscle mass, impaired cardiac and skeletal muscle energetics, and altered myocardial and whole‐body substrate metabolism (blunted fatty acid oxidation and elevated glucose metabolism) in individuals with BTHS [19, 20]; demonstrating a characteristic metabolic ‘phenotype' in BTHS. However, the effects of cardiac transplantation on the metabolic phenotype in BTHS is unknown. Therefore, the overall aim of this study was to compare ‘phenotypic’ information on skeletal morphology (fat and fat‐free mass), substrate metabolism, energetics and function (i.e., magnetic resonance imaging, exercise tolerance and cardiac function) in individuals with BTHS with cardiac transplantation to BTHS participants without transplantation and unaffected controls. Information gained from this study could advance our understanding of the metabolic and clinical presentation of patients with BTHS who have undergone cardiac transplantation and could provide insights into the pathophysiology of BTHS in the absence of overt cardiomyopathy.

## 2 Participants and Methods

### 2.1 Participants

Six individuals (*n* = 6) with BTHS who underwent cardiac transplantation (BTHS‐T) participated in the study, including *n* = 3 adolescents and 3 adults (Table 1). These participants were compared to data from individuals with BTHS without cardiac transplantation (BTHS‐noT, *n* = 29) and non‐affected healthy controls (Control, *n* = 28) who previously participated in a clinical exercise and metabolism phenotyping study [19] (NCT01625663). Inclusion criteria included (a) confirmed diagnosis of BTHS who underwent cardiac transplantation, (b) age 8–36 years, (c) sedentary (physically active less than 2×/wk), (d) medically stable and stable on medications for ≥ 3 months, and (e) residence in North America, the UK, Europe, or other locations with feasible travel to the U.S. Participants with BTHS were recruited via the Barth Syndrome Registry at the University of Florida. All participants underwent graded exercise testing, echocardiography, and clinical metabolism studies. However, body composition was not completed for 1 participant (air displacement plethymograph under repair) and clinical metabolism data (delay in breath sample analysis due to COVID‐19 led to ^13^CO_2_ breath sample decay in some participants) were only available for *n* = 3 due to technical issues. In addition, only *n* = 2 completed the MRS spectroscopy due to the presence of cardiac pacemakers.

**TABLE 1:** Participant demographics.

	Control (*n* = 28)	BTHS‐noT (*n* = 29)	BTHS‐T (*n* = 6)	*p*
Age (years)	17 ± 7	20 ± 8	18 ± 9	0.47
Height (cm)	166.0 ± 16.9	161.1 ± 20.2	149.4 ± 25.5	0.16
Weight (kg)	64.7 ± 22.3	52.0 ± 20.9	51.5 ± 23.1	0.076
BMI (kg/m^2^)	22.7 ± 5.2	19.1 ± 4.3*	23.7 ± 10.0	0.025
FFM (kg)	50.9 ± 16.8	35.6 ± 10.5*	40.7 ± 20.2	< 0.001
FFM (%)	80 ± 9	67 ± 11*	77 ± 5	< 0.001
FM (kg)	13.0 ± 8.3	19.6 ± 12.3*	11.9 ± 6.0	0.040
FM (%)	19 ± 8	33 ± 11*	23 ± 5	< 0.001
WBC (K/cumm)	5.4 ± 1.5	4.2 ± 1.9*	3.9 ± 0.8	0.010
Hemoglobin (g/dL)	13.6 ± 1.4	13.2 ± 1.3	12.6 ± 1.6	0.24
Hematocrit (%)	40.6 ± 4.1	38.8 ± 4.3	37.5 ± 5.0	0.13
PC (K/cumm)	214.0 ± 28.7	204.2 ± 56.4	250.8 ± 117.1	0.17
ANC (K/cumm)	3.0 ± 1.3	1.3 ± 0.9*	1.7 ± 1.1*	< 0.001
ALC (K/cumm)	1.8 ± 0.5	1.6 ± 0.4	1.2 ± 0.2*	0.040
AMC (K/cumm)	0.5 ± 0.2	0.8 ± 0.4*	0.7 ± 0.3	0.002
AEC (K/cumm)	0.20 ± 0.13	0.18 ± 0.10	0.30 ± 0.36	0.23
ABC (K/cumm)	0.01 ± 0.03	0.04 ± 0.07*	0.03 ± 0.05	0.049
Albumin (g/dL)	4.2 ± 0.2	3.8 ± 0.2*	3.7 ± 0.2*	< 0.001
Calcium (mg/dL)	9.2 ± 0.3	9.1 ± 0.4	8.9 ± 0.2	0.072
BUN (mg/dL)	14.5 ± 3.2	14.1 ± 4.3	19.3 ± 8.2*^§^	0.031
Bilirubin (mg/dL)	0.5 ± 0.4	0.4 ± 0.2	0.4 ± 0.1	0.12
AST (IU/L)	25.8 ± 21.2	27.1 ± 10.4	30.0 ± 7.8	0.84
ALT (IU/L)	20.5 ± 12.6	24.5 ± 13.4	20.5 ± 10.9	0.46
Creatinine (mg/dL)	0.7 ± 0.2	0.6 ± 0.2	0.8 ± 0.4	0.034
CO2 (mmol/L)	22.8 ± 2.3	22.5 ± 2.2	21.2 ± 1.2	0.27
Sodium (mmol/L)	137.9 ± 1.7	137.4 ± 2.2	137.8 ± 1.5	0.67
Total protein (g/dL)	6.7 ± 0.4	6.6 ± 0.4	6.7 ± 0.7	0.89
Potassium (mmol/L)	3.9 ± 0.2	4.0 ± 0.3	4.5 ± 0.4*^§^	< 0.001
Glucose (mg/dL)	89.5 ± 7.9	92.1 ± 11.1	89.8 ± 8.9	0.59
Insulin (μU/ml)	9.8 ± 6.4	12.0 ± 9.5	5.6 ± 1.6	0.21
TG (mg/dL)	78.4 ± 47.4	72.0 ± 34.6	64.8 ± 22.3	0.70
Total chol (mg/dL)	152.2 ± 25.6	117.7 ± 26.2*	102.8 ± 22.6*	< 0.001
HDL (mg/dL)	50.7 ± 11.3	43.5 ± 14.3	46.7 ± 14.7	0.13
LDL (mg/dL)	85.6 ± 24.0	59.7 ± 21.6*	43.5 ± 16.8*	< 0.001
IGF‐1 (ng/mL)	212.2 ± 66.9	125.1 ± 61.9*	92.6 ± 28.6*	< 0.001
Cortisol (ug/dL)	9.1 ± 2.6	13.1 ± 6.5*	12.2 ± 7.2	0.018

*Note:* Values are means ± SD. *p < 0.05 versus control, ^§^ *p* < 0.05 versus BTHS‐noT. Pairwise comparison *p* values adjusted for multiple testing using Tukey's Adjustment.

Abbreviations: ABC: absolute basophil count, AEC: absolute eosinophil count, ALC: absolute lymphocyte count, AMC: absolute monocyte count, ALT: alanine amino transferase, ANC: absolute neutrophil count, AST: aspartate amino transferase, BMI: body mass index, BUN: blood urea nitrogen, Chol: cholesterol, CO2: carbon dioxide, FFM: fat free mass, FM: fat mass, HDL: high‐density lipoprotein, IGF‐1: insulin‐like growth factor, LDL: low‐density lipoprotein, MPV: mean corpuscular volume, PC: platelet count, RBC: red blood cells, TG: triglyceride, WBC: white blood cells.

### 2.2 Ethical Approvals

The study was approved by the Human Research Protection Office at Washington University in St. Louis. Minor participants provided assent, and all participants and/or their guardians gave written informed consent.

### 2.3 Methods

All study procedures were conducted at the Washington University Institute for Clinical and Translational Sciences Clinical Research Unit (CTRU) over a two‐day period.

#### 2.3.1 Day 1: Study Visit Activities

##### 2.3.1.1 Body Composition

After a medical history review and physical exam by the study physician, fat mass (kg and %) and fat‐free mass (kg and %) were measured using air‐displacement plethysmography (Bod Pod, Life Measurements Inc., Concord, CA).

##### 2.3.1.2 Echocardiography

Participants underwent conventional 2D echocardiography, pulsed‐wave Doppler, and tissue Doppler imaging (General Electric Vivid E9, Waukesha, WI, USA). Left ventricular (LV) mass was assessed according to the American Society of Echocardiography guidelines [21]. LV end‐diastolic and end‐systolic volumes were measured using the method of discs, with ejection fraction calculated accordingly. LV dimensions were determined in the parasternal short‐axis view, and fractional shortening was computed as (LVED‐LVES)/LVED. 2D speckle tracking echocardiography was used to calculate global peak systolic strain by averaging measurements from the apical 4‐chamber, 2‐chamber, and long‐axis views.

##### 2.3.1.3 Skeletal Muscle Mitochondrial Energetics

Mitochondrial energetics in cardiac and skeletal muscle were assessed using ^31^Phosphorus Magnetic Resonance Spectroscopy (^31^P‐MRS) as previously described [22].

##### 2.3.1.4 Graded Exercise Testing

Participants performed a graded exercise test on a recumbent cycle ergometer (Lode, Netherlands). ECG, blood pressure, and oxygen consumption (V̇O_2_) were continuously monitored to determine peak exercise capacity (V̇O_2peak_, ParvoMedics, Sandy, UT). The protocol consisted of 1‐min incremental stages, starting at 10 W (BTHS groups) or 20 W (control group) and increasing by 10 or 20 W per minute until volitional exhaustion or symptom limitation.

#### 2.3.2 Evening Prior to Day 2

Participants consumed a standardized meal (12 kcal/kg body weight; 55% carbohydrate, 30% fat, 15% protein) prepared by the Bionutrition Research Kitchen at Washington University CRU. At 7:00 p.m., they drank a carbohydrate beverage (Boost; Nestle, Vevey, Switzerland) to replenish glycogen stores, then fasted (water allowed) from 8:00 p.m. until the study the next morning.

#### 2.3.3 Day 2: Study Visit Activities

##### 2.3.3.1 Substrate Metabolism Kinetics

Participants arrived fasted at 7:00 a.m. A catheter was placed in an antecubital vein for administering stable isotope‐labeled tracers, while another catheter in a contralateral hand vein (heated to 55°C) was used to obtain arterialized blood samples. Tracer infusions included ^13^C‐labeled sodium bicarbonate, [6,6‐^2^H_2_]glucose, and [U‐^13^C]palmitate, administered during a 210‐min study (120 min rest, 30 min exercise at 50% V̇O_2peak_, 60 min resting recovery). Blood and breath samples were collected for substrate and hormone analysis, as well as glucose and fatty acid kinetics. Whole‐body oxygen consumption (V̇O_2_) and carbon dioxide production (V̇CO_2_) were measured using indirect calorimetry (Parvo Medics, Sandy, UT). Plasma fatty acid oxidation rates were corrected for incomplete labeled CO_2_ recovery using established acetate correction factors [23].

### 2.4 Sample Analyses

Plasma Substrates and Hormones: Plasma glucose was measured with an automated analyzer (YSI 2300 STAT PLUS, Yellow Springs Instrument Co., Yellow Springs, OH). Plasma insulin and leptin were assessed via chemiluminescent immunometric methods (IMMULITEVR, Siemens, Los Angeles, CA). Free fatty acid concentrations were determined via gas chromatography (Hewlett‐Packard 5890‐II). Substrate kinetics and oxidation were determined as previously described [19].

### 2.5 Outcomes

The primary outcomes of this study were exercise tolerance (VO_2peak_, mL/kg/min), fat‐free mass (FFM, kg), glucose turnover during exercise (umol/kgFFM/min), plasma free fatty acid oxidation during exercise (umol/FFM/min), and skeletal muscle energetics (SM Qmax, mmol/s).

### 2.6 Statistical Analysis

Demographic and clinical characteristics of the cohort were reported stratified by cohort (BTHS‐T vs. BTHS‐noT vs. control). Categorical characteristics were summarized as frequency and percentage of non‐missing values. Normally distributed continuous characteristics were summarized as mean with standard deviation (SD), while skewed characteristics were reported as median with 25th and 75th percentiles (Q1, Q3), and range. Characteristics were compared among the three groups using analysis of variance (ANOVA) for continuous outcomes and Fisher's exact test for categorical outcomes.

To assess differences in exercise tolerance, body composition, metabolism, and energetics among BTHS‐T, BTHS‐noT, and control patients, generalized linear models were run with appropriate probability distributions and link functions assessed by residual analysis. Skewed outcomes (Plasma FFA oxidation and SM Qmax) were log‐transformed prior to running models. All models included age as a covariate.

Least‐squares (LS) means and LS mean differences (LSMD) or geometric means and geometric mean ratios (GMRs), if data were skewed, are reported comparing BTHS‐T to BTHS‐noT and BTHS‐T to control patients with 95% confidence intervals (CI). Boxplots were used to visualize differences in outcomes among the three groups.

## 3 Results

### 3.1 Demographics

Participant demographics are provided in Table 1. The average age of the cohort was similar among the three groups. At the time of the study, participants in BTHS‐T were taking the following medications: tacrolimus (*n* = 5), everolimus (*n* = 1), mycophenolate mofetil (*n* = 1), cyclosporine (*n* = 1), azathioprine (*n* = 1), amlodipine (*n* = 1), pravastatin (*n* = 3), aspirin (=4), vitamin D (*n* = 2), vitamin C (*n* = 1), vitamin B (*n* = 1), multivitamin (*n* = 2), acitretin (*n* = 1), atenolol (*n* = 1), montelukast (*n* = 2), folic acid (*n* = 1), fish oil (*n* = 1), iron (*n* = 1), coenzyme Q (*n* = 2), L‐citrulline (*n* = 1), prednisone (*n* = 1), fluticasone (*n* = 2), ranitidine (*n* = 1), fluxetine (*n* = 1), loratidine (*n* = 1), adderall XR (*n* = 1). Medications for BTHS‐NoT were previously reported [19]. For participants with BTHS who had cardiac transplantation, the mean time from cardiac transplantation to testing was 12.6 ± 5.7 years.

### 3.2 Body Composition

The average height of the cohort was relatively similar among the three groups; however, the average weight tended to be lower in the BTHS‐T and BTHS‐noT groups compared to the control, although this difference was not statistically significant (*p* = 0.076). BTHS‐noT patients had lower fat free mass in kilograms and as a percentage of their total mass compared to healthy controls, but slightly higher than BTHS‐noT patients; however, these differences did not meet statistical significance (Table 1).

### 3.3 Fasting Hormones and Metabolites

Plasma glucose, insulin, triglycerides, and high‐density lipoprotein were not significantly different between groups. Plasma total cholesterol and low‐density lipoprotein were significantly lower in both BTHS‐noT and BTHS‐T compared to control. Plasma cortisol was significantly higher in BTHS‐noT compared to control. Absolute neutrophil count and plasma albumin were significantly higher in BTHS‐T compared to BTHS‐noT and control (All Table 1).

### 3.4 Peak Exercise Testing and Cardiac Function

**FIGURE 1:**
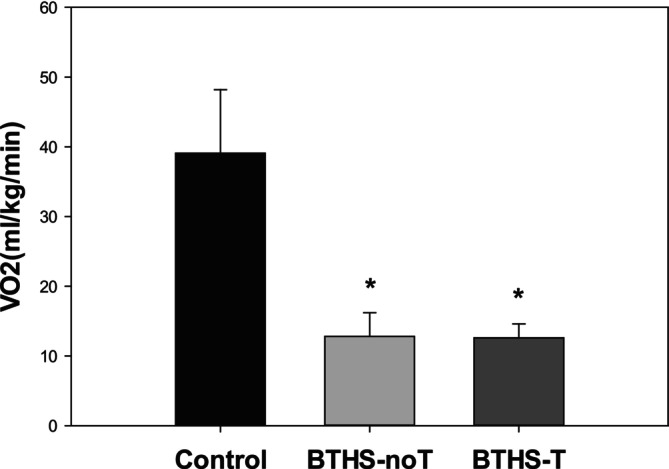
Volume of oxygen consumption at peak exercise (mean, SD, range). Control: participants without Barth syndrome (*n* = 28, 39.1 ± 9.1 (26.5, 65.2) mL/kgBW/min), BTHS‐noT: participants with Barth syndrome without cardiac transplantation (*n* = 29, 12.8 ± 3.4 (6.7, 19.9) mL/kgBW/min), BTHS‐T: participants with Barth syndrome with cardiac transplantation (*n* = 6, 12.6 ± 2.0 (11.3, 16.6) mL/kgBW/min). *p < 0.001 versus control.

Oxygen consumption at peak exercise (VO_2peak_) was significantly lower in both BTHS‐noT and BTHS‐T compared to control (Figure 1). In addition, respiratory exchange ratio (RER) at peak exercise was higher, and work rate and peak heart rate were lower in both BTHS‐noT and BTHS‐T compared to control (Table 2).

**TABLE 2:** Peak exercise and cardiac function.

Variables	Control	BTHS‐noT	BTHS‐T	Overall *p*
Peak exercise	(*n* = 28)	(*n* = 29)	(*n* = 6)	
Work rate (watts)	191.4 (70.3)	50.2 (15.1)*	35.8 (13.9)*	< 0.001
HR (bpm)	184.9 (9.7)	157.0 (17.3)*	144.5 (21.5)*	< 0.001
RER	1.1 (0.1)	1.4 (0.2)*	1.4 (0.2)*	< 0.001
Cardiac function	(*n* = 28)	(*n* = 29)	(*n* = 6)	
Resting HR (bpm)	71.0 (13.6)	80.6 (13.0)*	95.2 (12.2)*^§^	< 0.001
Resting SBP (mmHg)	123.7 (13.8)	102.0 (11.5)*	101.8 (8.1)*	< 0.001
Resting DBP (mmHg)	68.4 (8.1)	62.2 (9.8)*	67.0 (4.1)	0.048
FS (%)	37 (7)	34 (8)	35 (6)	0.55
EF (%)	65 (5)	60 (10)	60 (3)	0.065
S' septal	8.3 (1.4)	7.3 (1.9)	6.5 (1.2)	0.017
E' septal	13.5 (4.3)	11.3 (4.2)	11.7 (2.2)	0.16
E/E' septal	7.1 (1.9)	8.3 (3.2)	10.0 (3.1)	0.057
E/A	1.9 (0.4)	1.9 (0.7)	2.8 (0.5)*^§^	0.009
Global strain (%)	−19.7 (3.1)	−18.1 (3.5)	−17.9 (1.3)	0.17
Skeletal muscle energetics				
PCr time to recovery (Tau, s)	30.9 (7.1)	74.3 (22.8)*	68.4 (1.5)*	< 0.001
Qmax‐linear (mmol/s)	1.1 (0.3)	0.5 (0.1)*	0.4 (0.0)*	< 0.001

*Note:* Values are means ± SD, 95% CI. *p < 0.001 versus control. ^§^ *p* < 0.05 versus BTHS‐noT. Pairwise comparison *p* values adjusted for multiple testing using Tukey's Adjustment.

Abbreviations: bpm: beats per minute, DBP: diastolic blood pressure, E/A: early to late filling ratio during diastole, EF: ejection fraction, FS: fractional shortening, HR: heart rate, kgBW: kilogram of body weight, PCr: phosphocreatine, Qmax‐linear (maximum oxidative flux using the linear model), RER: respiratory exchange ratio (VCO_2_/VO_2_), SBP: systolic blood pressure [24].

At rest, heart rate was higher and systolic blood pressure was lower in both BTHS‐noT and BTHS‐T compared to control, and early to late (E/A) filling ratio was significantly higher in BTHS‐T compared to BTHS‐noT and control (Table 2).

### 3.5 Substrate Kinetics

**FIGURE 2:**
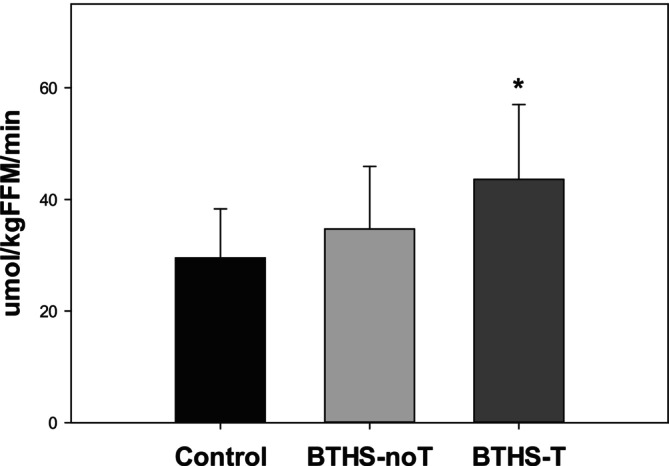
Glucose turnover rate during moderate intensity exercise (mean, SD, range). Control: participants without Barth syndrome (*n* = 28, 29.5 ± 8.8 (18.8, 56.3) μmol/kgFFM/min), BTHS‐noT: participants with Barth syndrome without cardiac transplantation (*n* = 29, 34.7 ± 11.2 (17.9, 61.7) μmol/kgFFM/min), BTHS‐T: participants with Barth syndrome with cardiac transplantation (*n* = 5, 43.6 ± 13.4 (23.9, 58.4) μmol/kgFFM/min). *p < 0.05 versus control.

**FIGURE 3:**
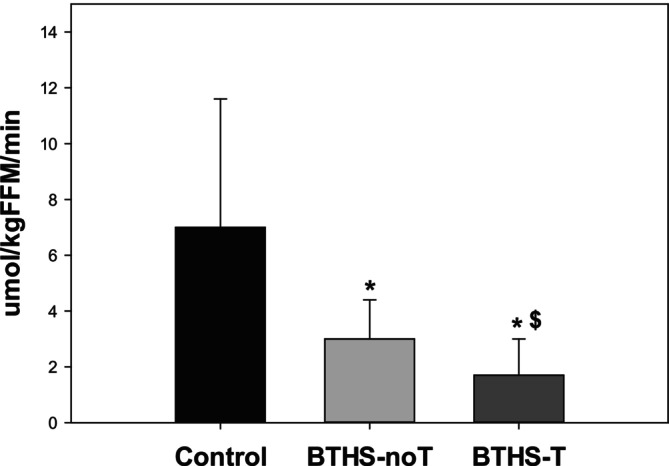
Free fatty acid oxidation rate during moderate intensity exercise (mean, SD, range). Control: participants without Barth syndrome (*n* = 28, 7.0 ± 4.6 (1.1, 20.5) μmol/kgFFM/min), BTHS‐noT: participants with Barth syndrome without cardiac transplantation (*n* = 29, 3.0 ± 1.4 (0.5, 6.4) μmol/kgFFM/min), BTHS‐T: participants with Barth syndrome with cardiac transplantation (*n* = 3, 1.7 ± 1.3 (0.3, 2.9) μmol/kgFFM/min). *p < 0.05 versus control, ^$^ *p* < 0.05 versus BTHS‐noT.

**FIGURE 4:**
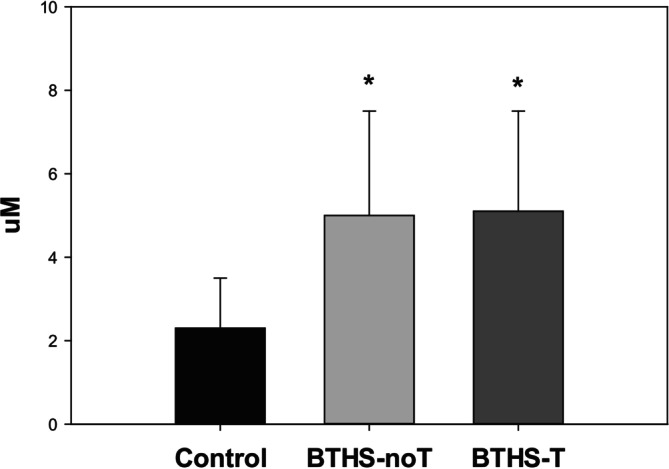
Plasma lactate concentrations during moderate intensity exercise (mean, SD, range). Control: participant without Barth syndrome (*n* = 28, 2.3 ± 1.2 (0.8, 4.8) μM), BTHS‐noT: participants with Barth syndrome without cardiac transplantation (*n* = 29, 5.0 ± 2.5 (1.3, 8.9) μM), BTHS‐T: participants with Barth syndrome with cardiac transplantation (*n* = 6, (3.0, 9.1) 5.1 ± 2.4 μM). *p < 0.05 versus control.

Adjusting for age, glucose turnover was significantly higher in BTHS‐T compared to control (LSMD: 12.30, 95% CI: 3.68–20.93; *p* = 0.005) but was not different from BTHS‐noT (LSMD: 5.67, 95% CI: −2.91 to 14.25; *p* = 0.20) (Figure 2). Plasma free fatty acid oxidation rate during exercise was slightly higher in BTHS‐noT compared to BTHS‐T adjusting for age (GMR: 2.32, 95% CI: 1.03–5.19; *p* = 0.041). Additionally, adjusting for age, control patients had a higher plasma free fatty acid oxidation rate compared to BTHS‐T and BTHS‐noT patients (GMR: 4.86, 95% CI: 2.18–10.83; *p* < 0.001) (Figure 3). Plasma lactate was significantly higher in both BTHS‐T and BTHS‐noT compared to control after adjusting for age (Figure 4).

### 3.6 Skeletal Muscle Energetics

Skeletal muscle phosphocreatine (PCr) time to recovery after exercise (Tau) was significantly slower and maximal oxidative flux (Qmax‐linear) was significantly higher among control patients compared to BTHS‐T patients and BTHS‐NoT patients (GMR: 2.54; 95% CI: 1.80–3.59 and GMR: 2.12, 95% CI: 1.86–2.42, respectively; *p* < 0.001 for both) adjusting for age. There was not a statistically significant difference between BTHS‐T and BTHS‐noT patients (all Table 2).

## 4 Discussion

Barth syndrome (BTHS) is a complex mitochondrial disorder primarily affecting cardiac function, skeletal muscle metabolism, and overall energy production [6]. Despite cardiac transplantation being a life‐saving intervention for many BTHS patients with end‐stage heart failure, it has equivalent post‐transplant survival and freedom from major post‐transplant outcomes to non‐BTHS recipients [25]; the broader metabolic consequences of heart transplantation remain poorly understood in this population. In this study, we compared the metabolic phenotype of individuals with BTHS who had undergone cardiac transplantation (BTHS‐T) to those with BTHS without transplantation (BTHS‐noT) and healthy controls. Our findings suggest that while cardiac transplantation may stabilize cardiac function, it does not restore body composition, skeletal muscle energetics, substrate utilization, or exercise tolerance to normal levels.

### 4.1 Exercise Tolerance and Skeletal Muscle Energetics

Cardiac transplantation is known to improve cardiac function in BTHS patients by alleviating heart failure symptoms and preventing further deterioration of cardiac output [26]. In addition, cardiac transplantation in non‐BTHS heart failure improves exercise tolerance [13, 14, 15, 16, 17] and skeletal muscle oxidative capacity [11]. However, our results indicate that BTHS‐T participants, despite improved cardiac function, still exhibited significantly lower peak oxygen consumption during exercise compared to control participants, indicating persistent exercise intolerance. Although we were only able to capture skeletal muscle energetics data in two participants with transplant, our finding is consistent with the persistence of impaired skeletal muscle energetics seen in these individuals, even after cardiac transplantation. Time to phosphocreatine recovery after exercise was significantly prolonged, and the maximal oxidative flux rate was reduced in BTHS participants [22], regardless of transplant status. This strongly suggests that even in the presence of improved cardiac function, skeletal muscle impairments in energy production severely limit exercise intolerance in BTHS. While this finding might not be totally unexpected due to the genetic nature of the skeletal muscle impairments in BTHS (and in other inherited cardioskeletal conditions), skeletal muscle oxidative capacity in non‐inherited heart failure is also impaired [24, 27] but is able to improve with cardiac transplantation [11, 14]. Lastly, fat‐free (i.e., skeletal muscle) mass was not significantly different among the three groups, in contrast to the finding of improved muscle mass following heart transplantation in those without BTHS [10]. The underlying cause of this skeletal muscle dysfunction is likely related to BTHS‐related mitochondrial abnormalities, specifically impaired cardiolipin remodeling in the inner mitochondrial membrane, which results in disrupted oxidative phosphorylation and compromised energy production in muscle cells [4]. Isolated soleus muscles from a mouse model of BTHS have also demonstrated significantly increased fatigability and reduced strength as compared to WT controls [28]. However, long‐term immunosuppressive therapy, a necessity for transplant recipients, could also contribute to muscle weakness and atrophy [24, 27] and may have affected the persistent exercise and skeletal muscle defects seen in our study.

### 4.2 Substrate Metabolism

One of the hallmark features of BTHS is a disruption in substrate metabolism, particularly with a blunted ability to oxidize fatty acids during exercise [19], which is essential for ATP production during activities with higher energy demand. Although we were only able to analyze data from *n* = 3 BTHS‐T participants, our data demonstrated that BTHS‐T exhibited significantly lower rates of fatty acid oxidation during exercise compared to both controls and BTHS‐noT groups. The reduction in fatty acid oxidation during exercise in all BTHS patients suggests that cardiac transplantation in BTHS may not improve fatty acid oxidation as seen after transplantation in non‐BTHS heart failure [18] and might indicate a continued increased reliance on glycolysis during exercise [19], which was reflected in the elevated lactate levels observed in both BTHS groups. Elevated lactate levels are a common feature of mitochondrial disorders, including BTHS [19], and can further contribute to exercise intolerance, as lactate accumulation impairs muscle function and recovery [29].

### 4.3 Implications for Treatment and Future Research

The results of this study have important clinical implications for the management of individuals with BTHS. Although cardiac transplantation can offer life‐saving benefits for BTHS patients with severe heart failure, it appears to not restore normal muscle function, exercise tolerance, or substrate metabolism. Our finding corroborates the patients' report that skeletal muscle weakness and exercise intolerance are their most significant symptoms and impact their daily life more than any other symptom [30]. Additionally, the findings emphasize the importance of early metabolic interventions and careful monitoring of skeletal muscle function in BTHS patients, even in those who undergo successful cardiac transplantation. Given the long‐term metabolic dysfunction observed in these patients, it is essential to identify additional treatments, especially strategies that may fully restore taffazin function in skeletal muscle, such as gene therapy. Transformative therapies that can address mitochondrial defects at the cellular level and mitigate the patient‐reported symptoms of chronic fatigue may substantially improve the overall quality of life for individuals living with BTHS.

## 5 Limitations

There were some limitations with this study. First, it was a small sample size and there were missing data due to technical difficulties, but overall BTHS is a rare disease and only 15% of patients undergo cardiac transplantation [8, 9]. Second, this was an observational study and we did not have pre‐transplant data. However, abnormalities in skeletal muscle mass, exercise tolerance, and substrate metabolism are consistent in individuals with BTHS [19, 31].

## 6 Conclusion

In conclusion, our study demonstrates that while cardiac transplantation can significantly improve heart function in BTHS, it does not restore skeletal muscle mass, energetics, substrate metabolism, or exercise capacity. The persistent metabolic abnormalities in both BTHS‐T and BTHS‐noT groups highlight the need for additional therapeutic approaches to address the skeletal muscle dysfunction in BTHS. These findings contribute to our understanding of the complex metabolic phenotype in BTHS and provide important insights for optimizing clinical management and treatment strategies in this rare disease.

## Author Contributions

W.T.C., K.L.B., B.J.B., C.T., B.F., and D.N.R. designed the study. W.T.C., K.L.B., L.d.l.F., L.R.P., A.B., and D.N.R. conducted the study. W.T.C., B.J.B., C.T., E.P., C.L.G., A.B., and D.N.R. analyzed and interpreted the data. W.T.C. wrote the manuscript. All authors edited the manuscript.

## Ethics Statement

Studies were approved by the Human Research Protection Office at Washington University in St. Louis.

## Consent

All minor participants provided assent and all participants and/or parents provided written informed consent.

## Conflicts of Interest

The authors declare no conflicts of interest.

## Data Availability

The data that support the findings of this study are available on request from the corresponding author. The data are not publicly available due to privacy or ethical restrictions.
